# Early recurrence after surgery in FIGO 2023 stage I-III endometrial cancer: characteristics and risk factors

**DOI:** 10.3389/fonc.2024.1500658

**Published:** 2025-01-06

**Authors:** Zhen Huang, Xue Li, Ling Li

**Affiliations:** ^1^ Department of Gynecology, The First Affiliated Hospital of Chongqing Medical University, Chongqing, China; ^2^ Department of Ultrasound, The First Affiliated Hospital of Chongqing Medical University, Chongqing, China

**Keywords:** endometrial cancer, early recurrence, LVSI, estrogen receptor, p53

## Abstract

**Background:**

Understanding the risk factors for early recurrence is crucial for improving endometrial cancer (EC) patient outcomes.

**Methods:**

We conducted a retrospective analysis of clinicopathological data from 473 patients diagnosed with EC at the First Affiliated Hospital of Chongqing Medical University between October 2013 and May 2019. We evaluated factors influencing early recurrence(defined as occurring within 12 months after treatment) based on 2023 International Federation of Gynecology and Obstetrics (FIGO) staging system.

**Results:**

Among the 473 patients, 284 (60.1%) were diagnosed with stage I, 117 (24.7%) with stage II, and 72 (15.2%) with stage III. A total of 343 patients (72.5%) had non-aggressive EC, while 130 patients (27.5%) had aggressive EC. Our findings identified higher FIGO stage, lymphovascular space invasion, estrogen receptor negativity, and abnormal P53 expression as significant independent risk factors for early recurrence. Of the 473 patients, 83 (17.6%) experienced recurrence, with 44 patients (53.0%) relapsing within 12 months post-treatment. Patients with early recurrence had significantly worse prognoses compared to those with late recurrence or no recurrence(P < 0.001).

**Conclusion:**

The identification of these risk factors is essential for developing individualized treatment plans and postoperative management strategies. Our study highlights the need for targeted therapies and intensified follow-up for high-risk patients to improve outcomes in endometrial cancer.

## 1 Introduction

Endometrial cancer (EC) is one of the most prevalent malignant tumors of the female reproductive system worldwide, with an increasing incidence, particularly in developed countries (1). Most patients with endometrial cancer present at early stages, typically due to abnormal vaginal bleeding (2). Following surgical resection of the tumor, along with comprehensive surgical staging and the addition of radiotherapy or chemotherapy, the overall recurrence rate of EC is approximately 15-20% (3–5). Given the large patient population, those who experience recurrence represent a significant group that cannot be overlooked.

Research indicates that the timing of recurrence is an independent prognostic factor for survival in patients with recurrent EC (6, 7). A shorter time to recurrence generally suggests higher malignancy, increased treatment difficulty, and an elevated risk of mortality (8). Early recurrence within one year after surgery is associated with decreased survival rates in EC patients (9, 10). Recurrence shortly after the completion of intensive treatment is understandably distressing for both patients and their families. Early recurrence not only adversely affects survival rates but also complicates treatment, often necessitating more invasive and aggressive adjuvant therapies, such as repeat surgeries, additional radiotherapy, and chemotherapy. Therefore, it is crucial for clinicians to understand and identify the high-risk factors for recurrence in this population.

Currently, studies on the factors influencing recurrence in EC predominantly focus on recurrences occurring 3-5 years post-treatment, with limited exploration of early recurrences, particularly those occurring within one year of treatment completion (11, 12). Furthermore, previous studies primarily utilized the 2009 International Federation of Gynecology and Obstetrics (FIGO) staging system for endometrial cancer, which has undergone significant revisions with the release of the updated 2023 FIGO staging system (13). Thus, this study aims to investigate the risk factors for early recurrence in endometrial cancer patients based on the 2023 FIGO staging system, with the goal of improving postoperative management and enhancing the quality of life for these patients.

## 2 Methods

### 2.1 Populations

We retrospectively collected clinicopathological data of patients diagnosed with endometrial cancer who underwent surgical treatment at the First Affiliated Hospital of Chongqing Medical University between October 2013 and May 2019. This study is a retrospective analysis conducted in accordance with the principles of the Declaration of Helsinki. The Ethics Committee of the First Affiliated Hospital of Chongqing Medical University approved this study (approval number: 2020-469). Considering the 2023 revision of the FIGO staging system, which updated the 2009 criteria to incorporate histological types, grading, and lymphovascular space invasion (LVSI), we reclassified the patients according to the new 2023 FIGO staging criteria11.

The inclusion criteria for the study were: 1) patients newly diagnosed with endometrial cancer who underwent standard comprehensive staging surgery, which included total hysterectomy with bilateral salpingo-oophorectomy, sentinel lymph node biopsy ± pelvic lymphadenectomy ± para-aortic lymphadenectomy; 2) patients with a postoperative pathological diagnosis of endometrial cancer; and 3) patients staged as FIGO 2023 stage I-III (patients with stage IV disease were excluded due to distant metastasis before surgery, which could affect recurrence assessment). The exclusion criteria were: 1) incomplete clinicopathological data; 2) loss to follow-up; and 3) presence of secondary malignancies.

### 2.2 Data collection

The following information was collected from the hospital’s electronic medical record system:1) Demographic information: age at cancer diagnosis, body mass index (BMI), and the presence of comorbidities (e.g., diabetes, hypertension). 2)Laboratory tests: preoperative serum levels of CA125 and HE4; 3) Pathological data: FIGO stage, histological subtype, LVSI, depth of lesion invasion, cervical involvement, and immunohistochemistry markers (Ki67, ER, PR, and P53).

According to the National Comprehensive Cancer Network (NCCN) guidelines for endometrial cancer, we defined advanced age as 60 years or older (5). Substantial LVSI was defined as ≥5 lymphovascular invasions on a single hematoxylin-eosin (HE) slide (5). The clinically recognized cutoff value for CA125 was 35 U/ml, while the HE4 cutoff was 70 pmol/L for premenopausal women and 140 pmol/L for postmenopausal women (14). Based on existing literature, ER and PR were considered positive when their expression exceeded 5% (15). And Ki67 was classified as high expression when more than 40% of cells were positive (16). Abnormal P53 expression was defined as either complete loss or overexpression, whereas normal P53 expression was defined as wild-type P53 (17).

### 2.3 Adjuvant treatment and follow-up

Following comprehensive surgical and pathological staging, the postoperative adjuvant therapy plan for patients was determined according to the most current international guidelines available at the time of diagnosis. Radiation therapy is generally recommended for patients with the following risk factors: age ≥60 years, non-endometrioid carcinoma, high-grade tumors (G3), deep myometrial invasion (≥1/2), cervical stromal invasion, LVSI positivity, pelvic lymph node involvement, or advanced FIGO stage. Specifically, combined chemotherapy is indicated for patients presenting with FIGO stage III, serous or clear cell carcinoma, G3 tumors, and deep myometrial invasion. Radiation therapy is typically initiated within 3 months post-surgery, which involves vaginal brachytherapy (total dose 22-45 Gy, 5.5-6 Gy/session, 2 sessions/week, for a total of 2 weeks with 4 sessions) or external pelvic irradiation (total dose 45-50 Gy, 1.8-2 Gy/session, 5 sessions/week, for 5 weeks with 25 sessions). The main chemotherapy regimen is the TP regimen (cisplatin and paclitaxel), administered every 3 weeks for 4-6 cycles. All patients received postoperative adjuvant therapy at our medical institution.

Patients were followed up every 3–6 months during the first 2 years post-treatment, every 6–12 months for the subsequent 3 years, and annually after 5 years. Follow-up assessments included a comprehensive physical and gynecological examination, inquiries about potential symptoms of recurrence, serum tumor marker tests, and imaging studies when clinically indicated. Recurrence was defined as either a tumor highly suspected based on imaging or confirmed by biopsy. Data on recurrence, including the time of onset, site of recurrence, and survival status, were recorded.

Early recurrence was defined as occurring within 12 months after the completion of treatment, while late recurrence referred to recurrence occurring more than 1-year post-treatment. Local recurrence was defined as a lesion located in the vagina or pelvis. Distant recurrence included metastases to para-aortic lymph nodes, abdominal metastases, or metastases to solid organs such as the lungs, liver, or bones. Follow-up continued until May 2024. Recurrence-free survival (RFS) was defined as the time from surgical resection to the date of recurrence. Overall survival (OS) was defined as the time from surgical resection to death or the last follow-up.

### 2.4 Statistical analysis

Statistical analyses and graphing were performed using SPSS version 26.0 and R software. Continuous variables were expressed as mean ± standard deviation or median ± Interquartile Range (IQR), while categorical variables were presented as frequencies and percentages. Group comparisons were conducted using one-way analysis of variance (ANOVA), the Kruskal-Wallis test, the Chi-square test, the t-test, and the Mann-Whitney U test. Kaplan-Meier curves were used for survival analysis, with the log-rank test employed to compare differences in survival times. Collinearity among all clinicopathological factors was assessed using tolerance and variance inflation factor (VIF) values. A univariate Cox regression model was used to evaluate risk factors affecting recurrence-free survival (RFS) in patients with early recurrence of endometrial cancer, and factors with a P-value <0.05 were further included in a multivariate Cox regression model. A two-sided P-value <0.05 was considered statistically significant.

## 3 Results

### 3.1 Patients characteristics

Based on the inclusion and exclusion criteria, 473 patients were included in this study. **Table 1** compared the clinicopathological characteristics of patients with no recurrence, early recurrence and late recurrence. Of the 473 patients, 284 (60.1%) were diagnosed with stage I, 117 (24.7%) with stage II, and 72 (15.2%) with stage III. 343 patients (72.5%) had non-aggressive EC, while 130 patients (27.5%) had aggressive EC. In addition, 289 patients (61.1%) had normal P53 expression, whereas 184 patients (38.9%) had abnormal P53 expression. Among the 473 patients, 83 (17.6%) experienced recurrence. Of those, 44 patients (53.0%) had recurrence within 12 months after treatment, with a mean age of 56.02 ± 9.25 years. The remaining 39 patients (47.0%) experienced recurrence after 12 months, with a mean age of 56.90 ± 8.95 years (P=0.016). Compared to patients without recurrence, those with recurrence had higher preoperative HE4 levels (P = 0.001) and more advanced FIGO stages (P < 0.001). No statistically significant differences were found among the three groups in terms of BMI (P = 0.100), hypertension (P = 0.200), diabetes (P = 0.799), or preoperative CA125 levels (P = 0.157).

**Table 1:** Characteristics of patients with no-, early- and late-recurrence (N=473).

Variables	No recurrence N=390 (%)	Early recurrence N=44 (%)	Late recurrence N=39 (%)	P-value
**Age** Mean ± SD Median (IQR)	53.21 ± 9.34 52 (47–60)	56.02 ± 9.25 54 (48-63)	56.90 ± 8.95 57 (50-63)	0.016
**BMI** Mean ± SD Median (IQR)	24.93 ± 3.68 24.45 (22.65-27.06)	23.70 ± 3.32 23.53 (21.18-26.26)	24.74 ± 2.75 24.97 (22.49-26.67)	0.100
**Hypertension** No Yes	297 (76.2%) 93 (23.8%)	35 (79.5%) 9 (20.5%)	25 (64.1%) 14 (35.9%)	0.200
**Diabetes** No Yes	335 (85.9%) 55 (14.1%)	38 (86.4%) 6 (13.6%)	32 (82.1%) 7 (17.9%)	0.799
**Pre-CA125** Mean ± SD Median (IQR)	45.57 ± 116.28 21.2 (13.7-34.68)	74.92 ± 90.37 31.5 (16.33-99.14)	31.34 ± 26.56 20.4 (14.0-36.0)	0.157
**Pre-HE4** Mean ± SD Median (IQR)	72.95 ± 66.76 56 (43-78)	141.11 ± 136.69 54 (47-99)	103.64 ± 120.60 70 (55-103)	0.001
**Lymphadenectomy** Yes No	349 (89.5%) 41 (10.5%)	38 (86.4%) 6 (13.6%)	34 (87.2%) 5 (12.8%)	0.764
**FIGO 2023 Stage** **I** I A1 I A2 I A3 IB **II** II A II B II C **III** III A III B III C	266 (68.2%) 121(31.0%) 47(12.0%) 49(12.6%) 49(12.6%) 88 (22.6%) 33(8.5%) 18(4.6%) 37(9.5%) 36 (9.2%) 18(4.6%) 7(1.8%) 11(2.8%)	6 (13.6%) 1(2.3%) 0(0%) 2(4.5%) 3(6.8%) 9 (20.5%) 0(0%) 4(9.1%) 5(11.4%) 29 (65.9%) 7(15.9%) 8(18.2%) 14(31.8%)	12 (30.8%) 3(7.7%) 3(7.7%) 4(10.3%) 2(5.1%) 20 (51.3%) 2(5.1%) 12(30.8%) 6(15.4%) 7 (17.9%) 2(5.1%) 3(7.7%) 2(5.1%)	<0.001
**Cervical stromal Invasion** No Yes	335 (85.9%) 55 (14.1%)	22 (50%) 22 (50%)	24 (61.5%) 15 (38.5%)	<0.001
**Invasion Depth** <1/2 myometrium ≥1/2 myometrium	291 (74.6%) 99 (25.4%)	17 (38.6%) 27 (61.4%)	22 (56.4%) 17 (43.6%)	<0.001
**LVSI** No or Focal Substantial^#^	354 (90.8%) 36 (9.2%)	26 (59.1%) 18 (40.9%)	23 (59.0%) 16 (41.0%)	<0.001
**Histology Type** non-Aggressive^*^ Aggressive^**^	295 (75.6%) 95 (24.4%)	22 (50.0%) 22 (50.0%)	26 (66.7%) 13 (33.3%)	0.001
**Ki67** Low High	305 (78.2%) 85 (21.8%)	23 (52.3%) 21 (47.7%)	23 (59.0%) 16 (41.0%)	<0.001
**Estrogen Receptor** Negative Positive	39 (10.0%) 351 (90.0%)	28 (63.6%) 16 (36.4%)	15 (38.5%) 24 (61.5%)	<0.001
**Progesterone Receptor** Negative Positive	56 (14.4%) 334 (85.6%)	27 (61.4%) 17 (38.6%)	17 (43.6%) 22 (56.4%)	<0.001
**P53** Normal Abnormal	247 (63.3%) 143 (36.7%)	20 (45.5%) 24 (54.5%)	22 (56.4%) 17 (43.6%)	0.058

^#^According to the 2023 FIGO for endometrial cancer, substantial LVSI is defined as the invasion of ≥5 blood vessels in one HE4 slide. ^*^Non-aggressive histology type: G1-2 endometrioid carcinoma. ^**^Aggressive histology type: high-risk histology types, including high-grade serous carcinoma, clear cell carcinoma, carcinosarcoma, dedifferentiated/undifferentiated carcinoma, and G3 endometrioid carcinoma.

### 3.2 Patterns of recurrence

**Table 2** provides data on the two groups of patients with recurrence. The median follow-up time for the 44 patients with early recurrence was 21 months (range: 7–107 months), with a median time to recurrence of 9 months (range: 2–12 months). Of the 44 patients, 30 (68.2%) presented with distant recurrence, including 5 with retroperitoneal lymph node recurrence, 9 with peritoneal recurrence, and 16 with recurrence in other organs. The remaining 14 patients (31.8%) exhibited local recurrence, including 6 with vaginal stump recurrence and 8 with pelvic region recurrence. For the 39 patients with late recurrence, the median follow-up time was 68 months (range: 20–108 months), with a median time to recurrence of 27 months (range: 14–50 months). Of the 39 patients, 17 (43.6%) developed distant metastasis, while 22 (56.4%) had local regional recurrence. Compared to patients with late recurrence, those with recurrence within 12 months after treatment had a higher proportion of distant metastases (P = 0.028) and a greater number of deaths, with statistically significant differences (P = 0.029).

**Table 2:** Recurrence characteristics and follow-up.

Patterns	Early Recurrence N=44 (%)	Late Recurrence N=39 (%)	P-value
**Recurrence** **Distant metastasis** Para-aortic lymph nodes Peritoneal Other organs **Local-regional** Vaginal stump Pelvic region	30 (68.2%) 5 9 16 14 (31.8%) 6 8	17 (43.6%) 4 6 7 22 (56.4%) 10 12	0.028
**RFS (months)** Mean ± SD Median (Range)	8.66 ± 2.79 9 (2-12)	28.00 ± 10.63 27 (14-50)	<0.001
**Dead** No Yes	15 (34.1%) 29 (65.9%)	23 (59.0%) 16 (41.0%)	0.029
**Follow-up (months)** Mean ± SD Median (Range)	37.07 ± 32.47 21 (7-107)	64.56 ± 10.63 68 (20-108)	<0.001

### 3.3 Overall survival analysis

**Figure 1:**
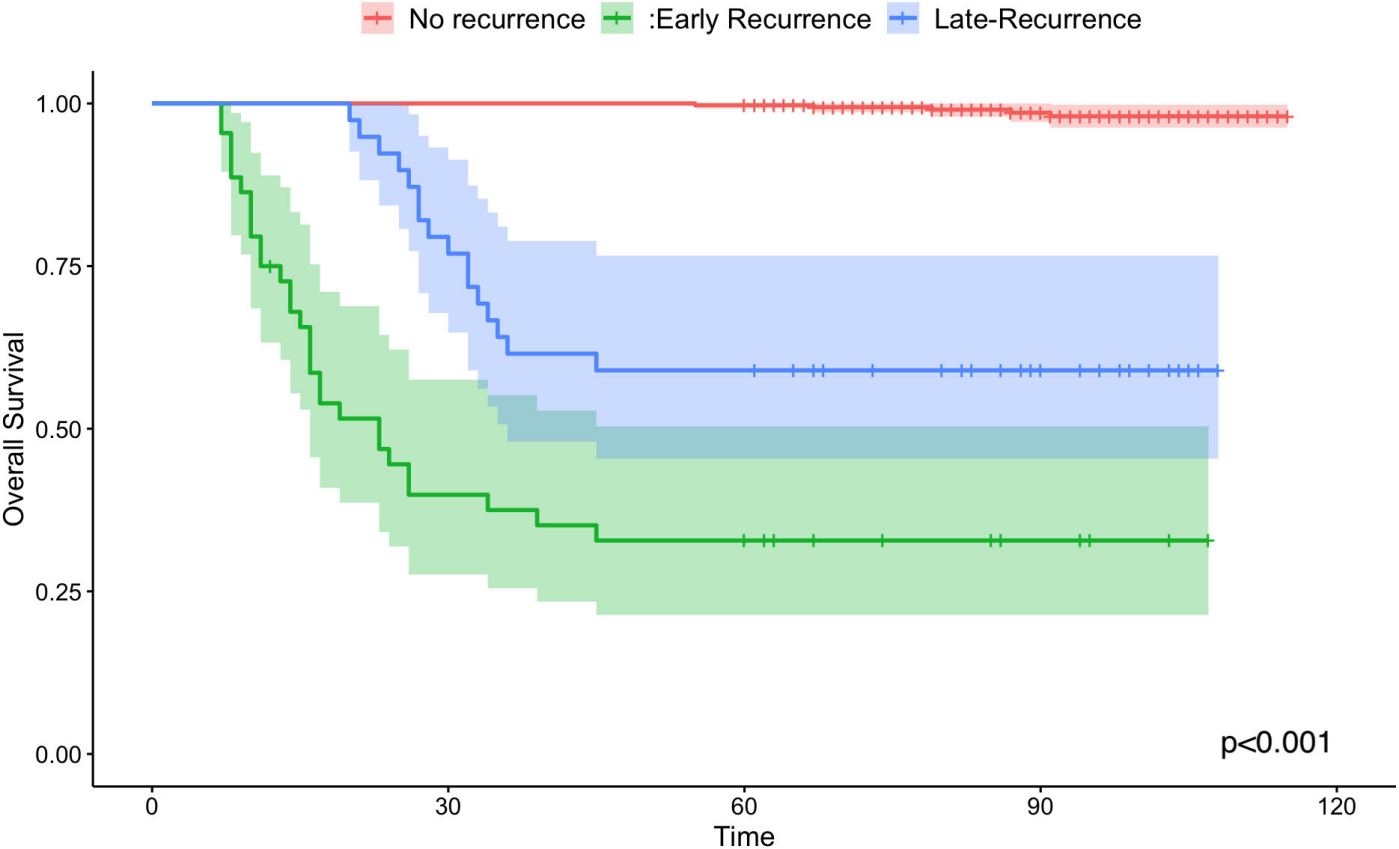
Kaplan-Meier survival curves of overall survival for no-recurrence (the red line), early recurrence group (the green line) and late recurrence group(the blue line).

Kaplan-Meier curves were employed to compare the overall survival rates among the no-recurrence, early recurrence, and late recurrence groups (shown in **Figure 1**). The 5-year overall survival rate was 98.7% (95% CI: 0.982-1.000) for patients without recurrence, 34.1% (95% CI: 0.223-0.517) for those with early recurrence, and 59.0% (95% CI: 0.454-0.766) for those with late recurrence. The log-rank test revealed a significant difference in overall survival among the three groups (P < 0.001).

### 3.4 Factors associated with early recurrence

First, we examined collinearity among the factors. **Supplementary Table 1** indicates that all factors included in the analysis had a tolerance greater than 0.1 and a VIF less than 5, suggesting no collinearity among the variables. Cox regression analysis was conducted to assess the factors influencing early recurrence in patients. Univariate Cox regression analysis revealed that age, BMI, diabetes, hypertension, and lymphadenectomy had P-values greater than 0.05 and were therefore excluded from the multivariate analysis. Factors with P-values less than 0.05 were subsequently analyzed using multivariate Cox regression.

The results indicated that higher FIGO stage (HR = 5.088, 95% CI: 1.537–16.842), the presence of LVSI (HR = 2.430, 95% CI: 1.227–4.813), ER negativity (HR = 0.294, 95% CI: 0.121–0.717), and abnormal P53 expression (HR = 2.422, 95% CI: 1.279–4.587) were independent factors influencing early recurrence after surgery in patients with endometrial cancer, all with statistically significant results. These results are presented in **Table 3**.

**Table 3:** Univariate and Multivariate analysis for early recurrence EC patients.

Variables	Univariate Analysis			Multivariate Analysis		
	HR	95%CI	p	HR	95%CI	p
**Age** (≥60 vs <60)	1.479	0.800-2.734	0.211			
**BMI** (≥24 vs <24)	0.711	0.394-1.285	0.259			
**Hypertension** (Yes vs No)	0.788	0.379-1.640	0.525			
**Diabetes** (Yes vs No)	0.932	0.394-2.206	0.874			
**CA125** (≥35 vs <35)	2.646	1.465-4.782	0.001	1.442	0.739-2.817	0.283
**HE4** (High vs Low)	1.412	0.679-2.937	0.356			
**Lymphadenectomy** (No yes Yes)	1.305	0.552-3.087	0.545			
**FIGO** I II III	1.000 3.725 24.126	- 1.326-10.466 10.006-58.171	<0.001 0.013 <0.001	1.000 1.126 5.088	- 0.341-3.723 1.537-16.842	0.001 0.845 0.008
**Cervical stromal Invasion** (Yes vs. no)	4.663	2.582-8.424	<0.001	1.390	0.691-2.794	0.356
**Invasion depth** (≥1/2 vs <1/2 myometrium)	3.950	2.153-7.248	<0.001	1.348	0.617-2.944	0.454
**LVSI** (Substantial^#^ vs. No or focal)	4.537	2.487-8.278	<0.001	2.430	1.227-4.813	0.011
**Histology type** (Aggressive^*^ vs Non-aggressive^**^)	2.789	1.544-5.036	0.001	1.511	0.797-2.867	0.206
**Ki67** (High vs. Low)	2.815	1.558-5.088	0.001	1.530	0.788-2.969	0.209
**ER** (Positive vs. Negative)	0.100	0.054-0.186	<0.001	0.294	0.121-0.717	0.007
**PR** (Positive vs. Negative)	0.149	0.081-0.273	<0.001	1.072	0.438-2.624	0.880
**P53** (Abnormal vs. Normal)	1.952	1.078-3.534	0.027	2.422	1.279-4.587	0.007

^#^According to the 2023 FIGO for endometrial cancer, substantial LVSI is defined as the invasion of ≥5 blood vessels in one HE4 slide. ^*^Non-aggressive histology type: G1-2 endometrioid carcinoma. ^**^Aggressive histology type: high-risk histology types, including high-grade serous carcinoma, clear cell carcinoma, carcinosarcoma, dedifferentiated/undifferentiated carcinoma, and G3 endometrioid carcinoma.

## 4 Discussion

Our findings indicate that patients with early recurrence exhibit distinct recurrence patterns and have significantly worse prognoses compared to those with late recurrence or no recurrence. Patients with a higher FIGO stage, presence of LVSI, ER negativity, and abnormal P53 expression are at an increased risk of early recurrence. Previous studies have reported that earlier recurrence of endometrial cancer is associated with a higher risk of mortality (7). Although a risk stratification system for EC has been established, there remains no consensus on the specific risk factors for early recurrence, particularly within the first year following treatment. Identifying these risk factors is crucial for clinicians in formulating individualized treatment plans, selecting appropriate adjuvant therapies, and developing postoperative follow-up strategies. For high-risk patients—especially those with FIGO stage III, LVSI positivity, ER negativity, and abnormal P53 expression, who are more likely to experience early treatment failure—more aggressive postoperative adjuvant therapies should be considered, such as extended cycles of radiotherapy and chemotherapy, as well as additional targeted or hormone therapies. Furthermore, more intensive postoperative follow-up is essential for reducing recurrence rates and improving survival outcomes.

This study identified differences in the recurrence patterns between early and late recurrence groups. Patients with early recurrence had a higher proportion of distant metastasis, whereas those with late recurrence exhibited a greater incidence of local recurrence. These findings suggest that patients with early recurrence may benefit from systemic therapy and close monitoring for distant metastasis, while patients with late recurrence may require prolonged local surveillance. One study reported that for advanced endometrial cancer, particular attention should be given to the primary site of the disease, as the risk of recurrence at this site is elevated (18). Future research should focus on elucidating the molecular drivers of different recurrence patterns to develop targeted interventions and optimize follow-up strategies.

As the FIGO stage increases, the risk of early recurrence significantly rises, which is consistent with the natural course and progression of endometrial cancer. Higher FIGO stages are associated with increased tumor aggressiveness and an increased likelihood of tumor cells spreading to lymph nodes or distant organs, thereby elevating the risk of recurrence (19). Patients in stage I have a lower risk of recurrence, while those in stage III—where the tumor may have already invaded surrounding tissues or lymph nodes—show a significantly higher recurrence rate. This underscores the importance for clinicians to pay particular attention to the recurrence risks in stage III patients when developing postoperative treatment plans and considering more aggressive adjuvant therapies.

Additionally, we found that lymphovascular space invasion is another independent risk factor for early recurrence. The presence of LVSI indicates that tumor cells have likely entered the lymphatic or vascular systems, increasing the likelihood of distant metastasis and recurrence (20). Numerous previous studies have supported the significant association between LVSI and recurrence (21, 22). The inclusion of LVSI in the updated FIGO staging system for endometrial cancer underscores its importance in predicting recurrence (13).

Hormone receptor status plays a critical role in the recurrence of endometrial cancer. Our study found that ER-negative patients had a higher risk of recurrence compared to ER-positive patients. The expression of estrogen receptors (ER) is closely linked to the degree of tumor differentiation and aggressiveness (23, 24). ER-positive patients typically respond more favorably to hormone therapy, leading to relatively better prognoses (25, 26). In contrast, ER-negative patients, due to their lack of response to hormone therapy, generally face a higher risk of recurrence (27).

P53 abnormalities were also identified in our study as a risk factor for early recurrence. P53 is a key tumor suppressor gene, and its mutations are often associated with high aggressiveness and poor prognosis (28). Mutations in P53 may allow tumor cells to evade normal cell cycle regulation, increasing their invasiveness and metastatic potential (29). Therefore, P53 status could serve as an important molecular marker for assessing the risk of recurrence in endometrial cancer (30). In recent years, international guidelines have recommended integrating molecular profiling into the risk stratification of EC (31). The molecular subtypes of EC include p53 mutant (p53-mut), POLE-mutated (POLE mut), mismatch repair-deficient (dMMR), and no specific molecular profile (NSMP). However, the high costs and time-consuming nature of genetic testing have limited the application of molecular profiling in certain regions and healthcare institutions. Recent studies have shown that new algorithms combining molecular classification with clinical and pathological features can help guide the assessment of EC recurrence risk, thereby saving resources while still providing valuable guidance for the selection of postoperative adjuvant therapies (32, 33). In the future, P53-targeted therapies may offer more effective treatment options for patients at high risk of recurrence.

It is important to note that while our study suggests that only FIGO stage, LVSI, ER negativity, and P53 status are independent risk factors for early postoperative recurrence in endometrial cancer patients, we do not deny the significance of other factors. For example, lymph node metastasis is a key prognostic factor for EC patients (34). Some studies have found that systematic lymphadenectomy does not necessarily provide survival benefits for early-stage EC patients and may even lead to some adverse treatment reactions (35). In recent years, sentinel lymph node biopsy has gained widespread attention and application. Compared to pelvic lymphadenectomy, it offers higher sensitivity and specificity for detecting lymph node metastasis, while maintaining good safety (36).

To the best of our knowledge, our study may be the first to focus on recurrence within one year after surgery in endometrial cancer patients. However, it is important to note that our study also has some limitations. First, this is a single-center retrospective study, and the relatively small sample size of the included patients may introduce selection bias. Second, proteins such as MMR, L1CAM, and CTNNB1 have recently been reported to be associated with the prognosis of endometrial cancer, and the molecular classification of endometrial cancer proposed by TCGA has also gained wide attention (37). Additionally, certain non-coding RNAs (ncRNAs) play a significant role in the development and progression of EC and may serve as potential predictors of patient prognosis (38). However, due to the retrospective nature of this study, we did not include these factors, and only considered P53 status from the molecular profiling. Large-scale, multicenter prospective studies in the future will help further validate our findings and provide data to support the development of more precise recurrence models. Thirdly, we did not report the treatment for recurrence, which can affect overall survival. Lastly, our study cohort consisted of all pathological types of endometrial cancer patients with FIGO 2023 stages I-III, without further stratification. In fact, patients with endometrioid carcinoma and non-endometrioid carcinoma have different recurrence risks, and early-stage patients (stages I-II) and advanced-stage patients (stage III) also have different recurrence risks. Future research should focus on the early recurrence risk in specific subgroups.

In conclusion, our findings suggest that early recurrence of endometrial cancer after surgery is closely related to FIGO stage, LVSI, ER negativity, and P53 status. Our study contributes to more accurate risk stratification and decision-making in clinical practice. Future research should focus on these risk factors to optimize treatment strategies and prolong patients’ disease-free survival.

## Data Availability

The datasets used and analyzed during the current study are available from the corresponding author on reasonable request.
