# Supplementary material for: Early recurrence after surgery in FIGO 2023 stage I-III endometrial cancer: characteristics and risk factors
Source: Front Oncol. 2025 Jan 6;14:1500658. doi: 10.3389/fonc.2024.1500658 (PMC11743483; doi:10.3389/fonc.2024.1500658)
Supplement: Supplementary file 1 [file Table1.docx]

**Supplementary Table 1 Collinearity Analysis of varibles**

| **Variables** | **Tolerance** | **VIF** |
| --- | --- | --- |
| **Age** | 0.865 | 1.156 |
| **BMI** | 0.924 | 1.082 |
| **Hypertension** | 0.857 | 1.166 |
| **Diabetes** | 0.896 | 1.116 |
| **preoperative CA125** | 0.824 | 1.214 |
| **preoperative HE4** | 0.848 | 1.179 |
| **FIGO** | 0.410 | 2.436 |
| **Lymphadenectomy** | 0.951 | 1.051 |
| **cervical stromal invasion** | 0.650 | 1.538 |
| **myometrial invasion** | 0.794 | 1.260 |
| **LVSI** | 0.773 | 1.293 |
| **Histology** | 0.857 | 1.166 |
| **Ki67** | 0.893 | 1.120 |
| **Estrogen Receptor** | 0.515 | 1.940 |
| **Progesterone Receptor** | 0.501 | 1.995 |
| **P53** | 0.969 | 1.032 |
